# The therapeutic effect and mechanism of parthenolide in skeletal disease, cancers, and cytokine storm

**DOI:** 10.3389/fphar.2023.1111218

**Published:** 2023-03-09

**Authors:** Sipin Zhu, Ping Sun, Samuel Bennett, Oscar Charlesworth, Renxiang Tan, Xing Peng, Qiang Gu, Omar Kujan, Jiake Xu

**Affiliations:** ^1^ Department of Orthopaedics, The Second Affiliated Hospital and Yuying Children's Hospital of Wenzhou Medical University, Wenzhou, Zhejiang, China; ^2^ School of Biomedical Sciences, The University of Western Australia, Perth, WA, Australia; ^3^ Department of Endocrinology, The First Affiliated Hospital of Guangdong Pharmaceutical University, Guangzhou, Guangdong, China; ^4^ The State Key Laboratory of Pharmaceutical Biotechnology, Institute of Functional Biomolecules, Nanjing University, Nanjing, China; ^5^ Research Center for Drug Discovery, School of Pharmaceutical Sciences, Sun Yat-sen University, Guangzhou, China; ^6^ UWA Dental School, The University of Western Australia, Perth, WA, Australia

**Keywords:** parthenolide, skeletal diseases, cancers, inflammation, osteolysis

## Abstract

Parthenolide (PTL or PAR) was first isolated from Magnolia grandiflora and identified as a small molecule cancer inhibitor. PTL has the chemical structure of C15H20O3 with characteristics of sesquiterpene lactones and exhibits the biological property of inhibiting DNA biosynthesis of cancer cells. In this review, we summarise the recent research progress of medicinal PTL, including the therapeutic effects on skeletal diseases, cancers, and inflammation-induced cytokine storm. Mechanistic investigations reveal that PTL predominantly inhibits NF-κB activation and other signalling pathways, such as reactive oxygen species. As an inhibitor of NF-κB, PTL appears to inhibit several cytokines, including RANKL, TNF-α, IL-1β, together with LPS induced activation of NF-κB and NF-κB -mediated specific gene expression such as IL-1β, TNF-α, COX-2, iNOS, IL-8, MCP-1, RANTES, ICAM-1, VCAM-1. It is also proposed that PTL could inhibit cytokine storms or hypercytokinemia triggered by COVID-19 *via* blocking the activation of NF-κB signalling. Understanding the pharmacologic properties of PTL will assist us in developing its therapeutic application for medical conditions, including arthritis, osteolysis, periodontal disease, cancers, and COVID-19-related disease.

## Introduction

Parthenolide (PTL or PAR) has been widely used as a herbal medicine for various health conditions (Pareek et al., 2011). It was originally isolated from plants of the Asteraceae family during the 1970s, is known to contain sesquiterpene lactones (SLs), and has been used for the treatment of migraine, inflammation, arthritis, and tumors (Hall et al., 1980; Heinrich et al., 1998; Schinella et al., 1998). It has been well established that sesquiterpene, sharing a similar structural feature with lignans, diterpenes, triterpenes, and polyphenols and a biological property on an inhibitory effect of nuclear factor kappa B (NF-κB) activity (Nam, 2006).

NF-κB proteins exist in the cytoplasm in a complex with inhibitors of NF-κB (IκB), which are in an inactivated state, but become phosphorylated upon activation. They are subsequently ubiquitinated and degraded by proteasome-mediated pathways (May and Ghosh, 1997). This permits NF-κB proteins to be released from the complex and translocates to the nucleus (May and Ghosh, 1997), and regulate the transcription of a large number of genes required for inflammatory response, chemokine activation, cell adhesion and immune regulations (Nam, 2006).

**FIGURE 1:**
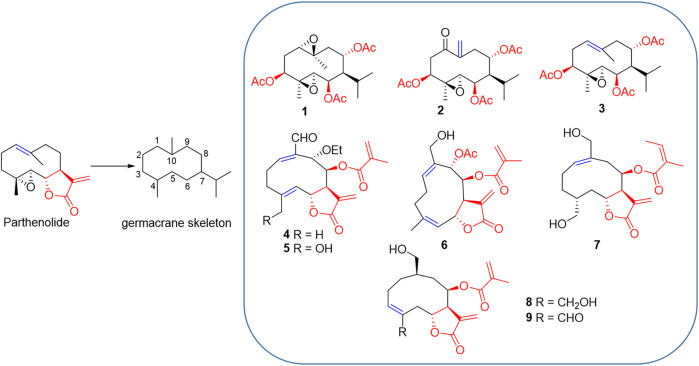
PTL and its analogues 1–9. Structural similarity: 1) they are all germacrane-type sesquiterpenoids sharing the same carbon skeleton (7-isopropyl-4,10-dimethylcyclodecane); 2) they all are highly oxygenated and have ester functionalities. Structural differences: 1) the configuration of carbon-carbon double bond (blue) is varied; 2) the types and substitutional positions of ester functionalities (red) are diverse.

PTL shares structural similarities with PTL analogues such as germacrane-type sesquiterpenoids, which bares the same carbon skeleton (7-isopropyl-4,10-dimethylcyclodecane) (Figure 1). They all are highly oxygenated with ester functionalities. In addition, they display structural differences, including the varied configurations of carbon-carbon double bond and diverse types and substitutional positions of ester functionalities (Figure 1). However, the relationship of these diverse chemical structures and their biological functions remains to be investigated.

Recent studies have shown the pharmacological effect of PTL on cancers, and structural modification of PTL could improve anticancer activity (Liu and Wang, 2022). In this review, we summarize the recent progress of the role of PTL and analogues in various conditions with emphasis on osteolytic diseases, primary and metastasis bone cancers, and COVID-19, which helps to fill the gap of knowledge through our investigation and discussion of the role of PTL and its derivatives in skeletal disease and cytokine storm. Understanding the pharmacological mechanisms of sesquiterpene lactone in the pathogenesis of various diseases will be important in developing an practical approach to prevent and treat these medical conditions.

## The role of PTL in skeletal disorders

Previous studies have shown that sesquiterpene lactone has anti-inflammatory effects (Schinella et al., 1998). It was subsequently revealed that it could block lipopolysaccharide (LPS)-induced osteolysis *via* the inhibition of osteoclast formation and bone resorption (Yip et al., 2004). Mechanically, PTL was found to inhibit NF-κB activity induced by pro-inflammatory cytokines such as tumour necrosis factor (TNF)-α, interleukin (IL)-1β, and receptor activator of nuclear factor kappa-Β ligand (RANKL), all of which have an inductive effect on osteoclast formation and activation (Yip et al., 2004). Further, PTL was found to attenuate polyethylene particles induced peri-implant osteolysis in a mouse model (Li et al., 2014; Zawawi et al., 2015). Consistently, PTL also was found to reduce empty lacunae and osteoclastic bone surface resorption induced by polyethylene particles in a murine calvarial model of peri-implant osteolysis for inflammation related orthopaedic aseptic implant loosening (Zawawi et al., 2015). Further, it prevents ovariectomy-induced bone loss *in vivo* by inhibiting osteoclast activity (Idris et al., 2010). The inhibition of osteoclast formation and bone resorption is vital to the therapeutic inhibition and prevention of osteolysis.

PTL consistently inhibits pro-inflammatory cytokine production and exhibits protective effects against the progression of collagen-induced arthritis in a rat model (Liu et al., 2015). PTL could inhibit ADAMTS and MMP—mediated degeneration of cartilage and osteoarthritis *via* its potential effect on chondrocytes (Legendre et al., 2005; El Mabrouk et al., 2007). At the cellular level, PTL can suppress LPS and TNF-α-induced increases in matrix metalloproteinase (MMP)-1, MMP-3, inducible nitric oxide synthase (iNOS), and IL-1β mRNA in chondrocytes (Liu et al., 2015), and inhibits TNF-α induced catabolism of aggrecan in cultured human chondrocytes (Zhou et al., 2008). During chemotherapy, it could also inhibit methotrexate (MTX)-induced osteoclastogenesis *via* attenuating TNF-α levels and the activation of NF-κB (King et al., 2012).

PTL and analogues have a protective effect on arthritis (Nam, 2006; Parada-Turska et al., 2008; Xu et al., 2009; Li et al., 2010; Liu et al., 2015). PTL inhibited the proliferation of rheumatoid arthritis fibroblast-like synoviocytes (RA-FLS) (Parada-Turska et al., 2008), as well as the expression of RANKL mRNA and protein in RA-FLS (Li et al., 2010). Interestingly, PTL has been found to attenuate neuropathy pains partly *via* the inhibition of intracellular signalling pathways NF-κB and MEK1/2 (Popiolek-Barczyk et al., 2014; Popiolek-Barczyk et al., 2015), which would be beneficial for arthritis related pain.

More recently, PTL was found to enhance alkaline phosphatase activity and mineralized nodule formation of osteoblasts in human periodontal ligament-derived cells (Zhang et al., 2017). These stimulated effect on osteoblasts were further evident by the increased expression of osteogenesis-related gene/protein expression of osteoblasts in the presence of TNF-α. Further, PTL inhibited the NF-κB/p50 pathway and resisted the inhibition of Wnt/beta-catenin signalling induced by TNF-α (Zhang et al., 2017). Interestingly, PTL increased cell viability and inhibited H2O2-induced apoptosis, indicating its role in inhibiting oxidative stress and cellular apoptosis in osteoblasts (Mao and Zhu, 2018). However, more recently, PTL was found to reduce the activity of ALP, alizarin red-positive mineralization, and the expression of ALP and osteocalcin mRNA using human periosteum-derived cells (hPDCs) (Park et al., 2020). In addition, PTL also attenuated the increased osteoblastic differentiation of TNF-α -treated hPDCs *via* the suppression of JNK signalling (Park et al., 2020). Consistently, PTL was previously found to have no inhibitory effects on osteoblast function using primary calvarial osteoblasts (Idris et al., 2010). The discrepancy observed in these studies might be due to the variation of cell types and culture conditions used, indicating the need for further research.

At the molecular level, PTL displays inhibitory effects on NF-κB, both at its transcriptional level and by direct inhibition of associated kinases, IKK-beta (IKK-β) (Mathema et al., 2012). Inhibition of NF-κB will influence the gene expression, including IL-1β, TNF-α, COX-2, iNOS, IL-8, MCP-1, RANTES, ICAM-1, and VCAM-1 (Nam, 2006). These molecules are critical for pro-inflammatory response, chemokine activation, cell adhesion and immune regulations (Nam, 2006).

NF-κB signaling is key to osteoclastogenesis. Previous studies have found that NF-κB subunits p50−/− and p52−/− null mice display osteopetrosis owing to lack of osteoclast formation (Franzoso et al., 1997; Iotsova et al., 1997). Conversely, activation of NF-κB resulted in excessive osteoclasts and osteolysis (Boyce et al., 1999; Xu et al., 2009). PTL appears to inhibit osteoclastogenesis and NF-κB activity induced by RANKL and LPS. For example, research suggests PTL is able to inhibit NF-κB activation by the attenuation of TNF-α induced IκB kinase complex activation (Hehner et al., 1999). Additionally, PTL and analogues appear to suppress NF-κB activity and RANKL-induced IκBα degradation (Yip et al., 2004; Qin et al., 2015).

In addition to its inhibitory role in the regulation of NF-κB, PTL was found to exert a biological effect as a phosphorylation inhibitor for signal transducer and activator of transcription (STAT1 and STAT3), and to prevent STAT1 and STAT3 DNA binding activity. Through this property, PTL can inhibit STAT-mediated transcriptional suppression of pro-apoptotic genes (Mathema et al., 2012).

More recently, PTL was found to inhibit inflammasome activity *via* the ATPase activity of nucleotide-binding oligomerization domain (NACHT), leucine rich repeat (LRR) and pyrin domain containing (PYD) 3 (NLRP3) (Juliana et al., 2010). NLRP3 is a member of the NACHT, LRR and PYD domains-containing protein 3 (NALP3) inflammasome complex, an upstream activator of NF-κB signalling. Through this action, PTL regulates the inflammatory response, immune regulation, and apoptosis. Collectively, PTL and sesquiterpene lactone compounds were able to affect NF-κB and other signalling molecules that results in osteoclast formation, bone resorption and osteolysis (Table 1).

**TABLE 1:** Therapeutic effects and cellular mechanisms of PTL.

Proposed therapeutic effects	Potentials cellular mechanisms	References
Skeletal and dental system		
LPS-induced osteolysis	Anti-osteoclastogenesis and bone resorption, anti-RANKL signalling	Yip et al. (2004), Qin et al. (2015)
Per-implant osteolysis	Anti-born resorption	Li et al. (2014), Zawawi et al. (2015)
Ovariectomy-induced osteoporisis	Anti-osteoclastic born resorption	Idris et al. (2010)
Methotrexate-induced bone loss	Anti-osteoclastogenesis and bone resorption, anti-inflammation	King et al. (2012)
Collagen-induced arthritis	Anti-inflammation in osteoclast-like cells and in chondrocytes	Liu et al. (2015)
Rheumatoid arthritis	Inhibit fibroblast-like synoviocytes (RA-FLS)	Parada-Turska et al. (2008), Mathema et al. (2012)
Periodontitis	Anti-inflammation in periodontal ligament-derived cells	Zhang et al. (2014), Zhang et al. (2017)
Cancers (related to skeletal system)		
Bone metastasis with breast cancer	Inhibit growth or promote apoptosis of W256 cells, anti-osteoclast	Idris et al. (2009), Marino et al. (2017)
Prostate cancer related osteolysis	Inhibit prostate cancer cells, mediate osteoclasts and osteoblasts	Marino et al. (2019)
Multiple myeloma	Anti-cancer stem cell activity, anti-TRAP6	Suvannasankha et al. (2008), Gunn et al. (2011), Kong et al. (2015)
Osteosarcoma	Induce autophagic cell death in osteosarcoma cells sensitize tumor	Zuch et al. (2012), Yang et al. (2016)
Osteosarcoma with lung metastasis	Inhibit cell proliferation and the expression of VEGF	Kishida et al. (2007)
Acute myeloid leukemia (AML)	Inhibit AML xenograft tumor growth, reverse drug resistance	Yip et al. (2004), Wang et al. (2006)
Oral cancer	Prevent tumor formation, chemopreventive, induce apoptosis	Yu et al. (2015), Baskaran et al. (2017)
Other cancers (as examples)		
Gastric cancer	Sensitize gastric cancer cells, reverse drug resistance	Li et al. (2018), Liu et al. (2020)
Pancreatic cancer	Suppresses pancreatic cancer cell growth, induce apoptosis	Yip-Schneider et al. (2005), Liu et al. (2010), Liu et al. (2017)
Hepatic cancer	Sensitize cancer cells, reverse drug resistance, induce apoptosis	Carlisi et al. (2011), Kim et al. (2012), Liu et al. (2013)
Lung cancer	Inhibit cancer cell growth, sensitize cancer cells	Li et al. (2020), Sun et al. (2020), Wu et al. (2020)
Ovarian cancer	Inhibit cancer cell growth and invasion, induce apoptosis	Lee et al. (2012), Takai et al. (2013), Kwak et al. (2014)
Glioblastoma	Suppresses cancer cell growth, induce apoptosis	Anderson and Bejcek (2008), Hexum et al. (2015), Ding et al. (2020)
Cytokine storm		
COVID-19	Anti-inflammation, immunomodulators	Bahrami et al. (2020), Soleymani et al. (2022)

## The role of PTL in cancers

NF-κB signalling pathway is constitutively activated in many types of cancer cells. Under physiological condition, inactive form of NF-κB is mainly bound to its inhibitor IκB and present in the cytoplasm. Upon activation by signalling molecules, IκBα is phosphorylated and degraded and releases NF-κB, which will transfer from the cytoplasm to the nucleus (Karin, 1999). Targeting NF-κB to reduce overexpression or activation of NF-κB, and its anti-apoptotic effect hint a role of PTL as a potential therapeutic target for the treatment of cancers (Fuchs, 2010), including gastric cancer (Li et al., 2018; Liu et al., 2020), pancreatic cancer (Yip-Schneider et al., 2005; Liu et al., 2010; Liu et al., 2017), hepatic cancer (Carlisi et al., 2011; Kim et al., 2012; Liu et al., 2013), lung cancer (Li et al., 2020; Sun et al., 2020; Wu et al., 2020), ovarian cancer (Lee et al., 2012; Takai et al., 2013; Kwak et al., 2014), glioblastoma (Anderson and Bejcek, 2008; Hexum et al., 2015; Ding et al., 2020), and oral cancer (Yu et al., 2015; Baskaran et al., 2017), just to name a few examples. In this review, we will focus on our discussion in more detail regarding the role of PTL in cancers related to the skeletal system (Table 1).

Research suggests that PTL could be a therapeutic agent for the treatment of osteosarcoma. PTL was found to sensitize radioresistant osteosarcoma cells and greatly reduce the prevalence of relapse and metastatic progression (Sugiyasu et al., 2011; Zuch et al., 2012). PTL also induced cell death in human osteosarcoma cells *via* reactive oxygen species (ROS)-mediated autophagy (Yang et al., 2016), and through caspase-independent and AIF-mediated signalling molecules (D'Anneo et al., 2013). In a murine animal model, PTL was able to inhibit lung colonization of osteosarcoma cells (Kishida et al., 2007). Taken together, these findings suggested that PTL through its inhibitory effect on NF-κB might serve as an antimetastatic drug.

In addition, PTL has potential effects on multiple myeloma (MM) (Suvannasankha et al., 2008; Gunn et al., 2011; Kong et al., 2015). It was found that PTL has anti-cancer stem cell activity (Gunn et al., 2011), as well as direct effects on MM cells and the bone marrow microenvironment in myeloma (Suvannasankha et al., 2008). The inhibitory effects of PTL in MM *via* targeting tumor necrosis factor receptor-associated factor 6 (TRAF6) and NF-κB pathways (Kong et al., 2015). PTL-induced apoptosis in MM cells involves ROS generation and cell sensitivity depends on catalase activity (Wang et al., 2006). In addition, PTL has inhibitory effects on angiogenesis induced by human MM cells (Kong et al., 2008).

PTL also has effects on cancers that are accompanied by bone osteolytic conditions such as breast cancer (Chen et al., 2009; Idris et al., 2009; Marino et al., 2017). It is therefore suggested that targeting NF-κB may be of value in the treatment of breast cancer related osteolysis (Marino et al., 2017), and the administration of PTL might be effective in preventing breast cancer mediated osteolysis (Chen et al., 2009; Idris et al., 2009).

Leukemia is often accompanied with severe bone loss due to the dysregulation in leukemia cells and bone microenvironment during leukemogenesis (Cheung et al., 2018; Anderson et al., 2020). PTL as a natural product has been suggested to target the leukemia stem cells (LSCs) in acute myeloid leukemia (AML) (Siveen et al., 2017) *via* regulating ROS levels and the anti-proliferative activity of cancer cells (Kempema et al., 2015). A panel of novel modified PTL analogues were found to possess significantly improved anti-leukemic potency against primary AML cells, with low toxicity against normal mature and progenitor hematopoietic cells *via* P450-mediated pathways (Kolev et al., 2014). PTL was found to have a radiosensitization effect in prostate cancer cells. Further, PTL inhibits NF-κB activity and the expression of phosphatase and tensin homologue deleted on chromosome 10 (PTEN) that is involved in the radiosensitization effect (Sun et al., 2007; Watson et al., 2009; Mendonca et al., 2017).

More recently, PTL was found to inhibit the growth of prostate cancer cells dose-dependently and reduce prostate cancer cell-osteoclast co-cultures mediated osteoclast formation, suggesting that PTL could reduce prostate cancer related osteolysis (Marino et al., 2019).

## The role of PTL in cytokine storm

Cytokines play a vital role in the homeostasis of the immune system. A cytokine storm could occur when a large number of cytokines are released in the body instantaneously, which may be life threatening and lead to multiple organ failure. A cytokine storm is also called hypercytokinemia, which is usually induced by an infection, autoimmune disorder, or other inflammatory disease.

A cytokine storm triggered by COVID-19 has been associated with respiratory failure and lung fibrosis (Cao, 2020; Yang et al., 2020; Lowery et al., 2021). It is evident that multiple inflammatory cytokines, including TNF-α, IL-1β, IL-6, and IL-10 play a significant role in the pathogenesis of COVID-19 induced morbidity and mortality (Peddapalli et al., 2021). For instance, increased IL-6 levels in circulating were tested in patients with cardiovascular conditions with a poor prognosis of COVID-19 (Clerkin et al., 2020; Guo et al., 2020). NF-κB signalling pathways are common to COVID-19 induced cytokine responses (Kircheis et al., 2020; Kandasamy, 2021; Peddapalli et al., 2021). The inhibition of NF-κB has therefore been proposed to be a target for the treatment of COVID-19 (Kircheis et al., 2020; Kandasamy, 2021; Kircheis et al., 2021). For example, propolis and digitoxin have been found to suppress levels of the cytokines and have benefits for the treatment of the comorbidities in COVID-19 patients (Berretta et al., 2020; Pollard et al., 2020).

In line with this, PTL, as a previously identified NF-κB inhibitor has been proposed to treat COVID-19 (Bahrami et al., 2020; Soleymani et al., 2022). Interestingly, using molecular docking, PTL analogues were found to binds with high affinity to the selected target of SARSCoV-2, which might serve as a potential candidate for anti-SARS-CoV-2 therapy (Ouled Aitouna et al., 2021; Lakhera et al., 2022). Consistently, feverfew which contains the major ingredient of PTL has long been used as a traditional medicine for the treatment of fever, migraine headache, and inflammatory conditions (De Weerdt et al., 1996; Koprowska and Czyz, 2010). More recently, PTL was found covalently bind to Cys-191 or Cys-194 of the coronavirus papain-like protease and inhibit its deISGylation and activity by allosteric regulation (Zou et al., 2022). Collectively, PTL is thought to be beneficial for the treatment of COVID-19 *via* the inhibition of cytokine storm with NF-κB signalling pathways activation (Bahrami et al., 2020; Ouled Aitouna et al., 2021; Soleymani et al., 2022) (Table 1).

## The role of PTL in periodontal disease

Periodontal disease is one the most common non-communicable diseases in humans, where nearly 1 billion people are affected (GBD, 2016 Disease and Injury Incidence and Prevalence Collaborators, 2017). The disease involves gingivitis and periodontitis and is caused by oral infection (Li et al., 2000). The current understanding of periodontal disease pathogenesis demonstrates host-related inflammatory responses triggered by bacterial pathogens whereby an influx of immune-host mediators such as prostaglandins, leukotrienes, complement activation products, chemokines, and cytokines are released to form a sophisticated network of interactions between elements of innate and adaptive immune systems (Hajishengallis et al., 2020). These complex interactions lead to inflammation-induced bone loss, primarily mediated by a triad of RANKL and NF-κB signalling pathway (Hajishengallis et al., 2020).

Recent research has explored the potential therapeutic effects of PTL for periodontal disease in both an *in vitro* model and on human alveolar bone tissue (Zhang et al., 2014; Zhang et al., 2017). PTL appears to inhibit the activation of major inflammatory pathways involved in periodontal disease *via* NF-κB and ERK signalling pathways, in addition to the expression of inflammatory and osteoclastogenic genes in lipopolysaccharide-stimulated human periodontal ligament cells (Zhang et al., 2014). Whilst PTL could also promote osteoblast differentiation *via* the Wnt/β-catenin signaling pathway and might be a pivotal target for periodontal bone regeneration (Zhang et al., 2017). Periodontitis would be a significant health burden if it was left without treatment. It will not only lead to teeth loss, but it can cause severe consequences to general health. The systemic-oral link is well established, and poorly controlled periodontitis can complicate the management of systemic conditions such as diabetes and cardiovascular diseases (Kane, 2017). PTL offers an excellent opportunity to enhance the current regimens in the management of periodontal disease, and further studies are required.

## Conclusion

**FIGURE 2:**
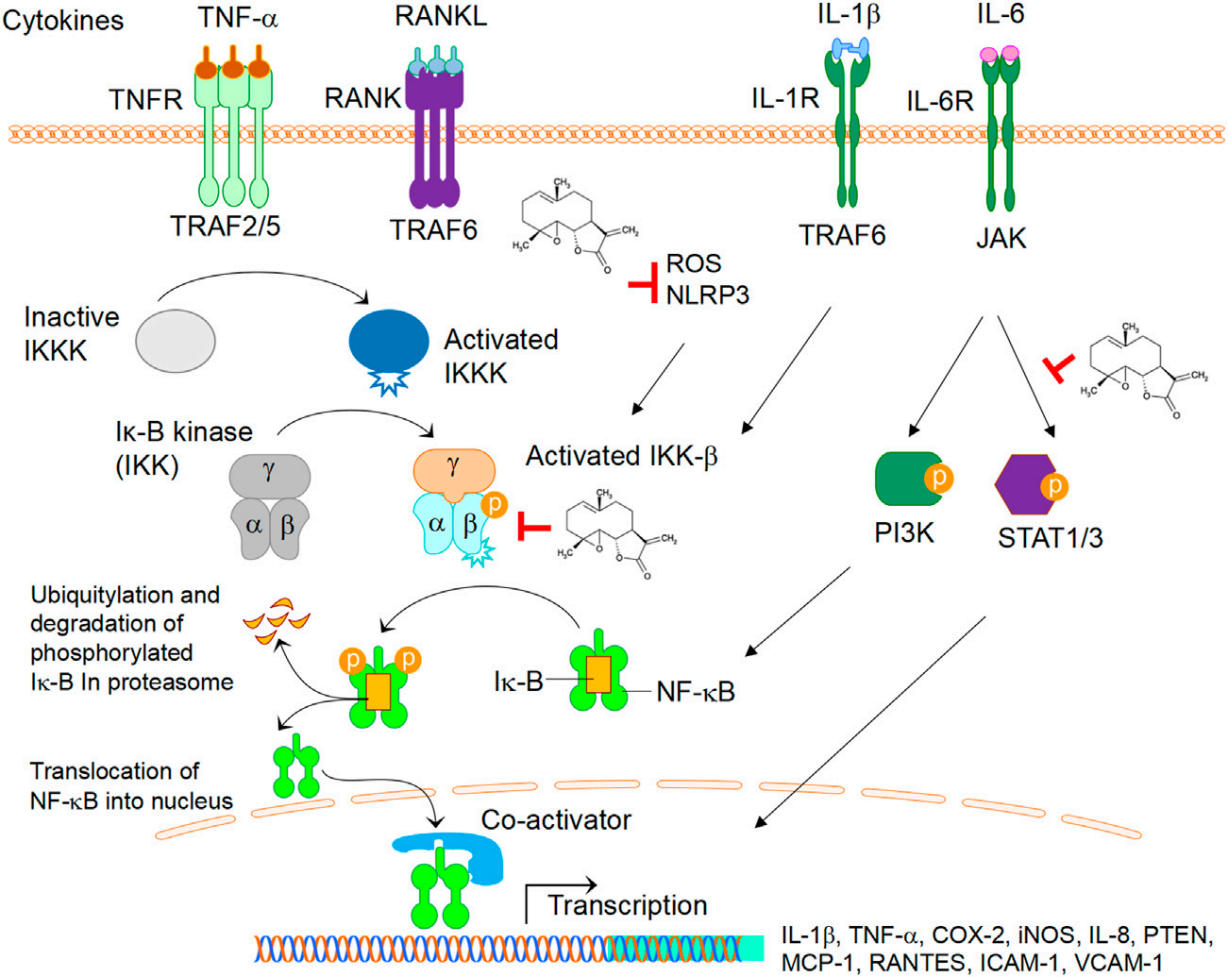
The role of PTL in cytokines-mediated signalling activation. Cytokines TNF-α, RANKL, IL-1β, IL-6, upon binding to their respective receptors, activate several signalling cascades leading to down-stream the transcriptions of genes such as IL-1β, TNF-α, COX-2, iNOS, IL-8, PTEN, MCP1, RANTES, ICAM, VCAM. Note that PTL displays inhibitory effects on NF-κB by direct inhibition of IKK-β, inflammasome activity *via* NLRP3, or ROS. PTL also act as a phosphorylation inhibitor for STAT1 and STAT3. Abbreviations: TNF-α, tumor necrosis factor alpha; RANKL, receptor activator of nuclear factor kappa-Β ligand; IL-1β, interleukin 1 beta; IL-6, interleukin 6; COX-2, cyclooxygenase-2; iNOS, inducible nitric oxide synthase; IL-8, interleukin 8; PTEN, phosphatase and tensin Homolog; MCP-1, monocyte chemoattractant protein-1; RANTES, regulated upon activation, normal T cell expressed and secreted; ICAM-1, intercellular adhesion molecule 1; VCAM-1, vascular cell adhesion molecule 1.

In short, PTL has therapeutic effects on skeletal diseases, primary and metastasis bone cancers, and inflammation-induced cytokine storm. As summarised in Figure 2, PTL displays inhibitory effects on cytokine-mediated NF-κB by direct inhibition of IKK-β (Mathema et al., 2012), or indirect inhibitory effects on inflammasome activity *via* NLRP3 (Juliana et al., 2010) and ROS (Kempema et al., 2015) (Figure 2). PTL also act as a phosphorylation inhibitor for STAT1 and STAT3 (Mathema et al., 2012) (Figure 2). Further understanding the mechanistic insights into the role of PTL in a disease specific manner will enable us to develop therapeutic applications of PTL for arthritis, osteoporosis, periodontal disease, cancer, and COVID-19. In addition, there are limitations regarding the clinical use of PTL, including pharmacological doses, routes of drug delivery and double-blind clinical trial studies, and future studies in addressing these issues will enhance the clinical applications of PTL in various diseases. Previous studies have focused on the efficacy of PTL in various conditions with a lack of reports on toxicity assessments. As a general rule, since PTL is an NF-κB inhibitor, it might cause liver toxicity when used to overdose. Thus, further systemic evaluation of liver toxicity will help to better understand the druggability of PTL.
